# GuitarPD: A Randomized Pilot Study on the Impact of Nontraditional Guitar Instruction on Functional Movement and Well-Being in Parkinson's Disease

**DOI:** 10.1155/2022/1061045

**Published:** 2022-06-25

**Authors:** Serap Bastepe-Gray, Lavinia Wainwright, Diane C. Lanham, Gabriela Gomez, Jieung S Kim, Zane Forshee, Bonnie Kaas, Arita McCoy, Colin McGregor, Emile Moukheiber, Suraj Rajan, Gerson Suarez-Cedeno, Jiangxia Wang, Sean Brennan, Shane Coughlin, Kyurim Kang, Alexander Pantelyat

**Affiliations:** ^1^Department of Neurology, Johns Hopkins University School of Medicine, Baltimore, MD, USA; ^2^Peabody Conservatory, Johns Hopkins University Peabody Institute, Baltimore, MD, USA; ^3^Department of Biostatistics, Johns Hopkins University School of Public Health Biostatistics Center, Baltimore, MD, USA

## Abstract

Playing musical instruments may have positive effects on motor, emotional, and cognitive deficits in patients with Parkinson's disease (PD). This pilot study examined the feasibility of a six-week nontraditional guitar instruction program for individuals with PD. Twenty-six participants with idiopathic PD (Age: 67.22 ± 8.07; 17 males) were randomly assigned to two groups (intervention first or 6 weeks of usual care control exposure) with stepwise exposure to the guitar intervention condition with cross-over at six weeks. Outcomes were assessed at baseline, 6, 12, and 18 weeks. Twenty-four participants completed the study. Combined analysis of the groups showed significant BDI-II improvement immediately after intervention completion (3.04 points, 95% CI [−5.2, −0.9], *p* = 0.04). PDQ-39 total quality of life scores improved from baseline to immediately postintervention 5.19 points (95% CI [−9.4, −1.0]) at trend significance (corrected *p* = 0.07). For Group 1 (exposed to the intervention first), MDS-UPDRS total scores improved by a mean of 8.04 points (95% CI [−12.4, −3.7], *p* = 0.004) and remained improved at 12 weeks by 10.37 points (95% CI [−14.7, −6.0], *p* < 0.001). This group also had significant improvements in mood and depression at weeks 6 and 12, remaining significant at week 18 (BDI-II: 3.75, 95% CI [−5.8, −1.7], *p* = 0.004; NeuroQoL-depression: 10.6, 95% CI [−4.9. −1.4], *p* = 0.004), and in anxiety at week 6 and week 18 (NeuroQoL; 4.42, 95% CI [−6.8, −2.1], *p* = 0.004; 3.58, 95% CI [−5.9, −1.2], *p* = 0.02, respectively). We found clinically and statistically significant improvements in mood/anxiety after 6 weeks of group guitar classes in individuals with PD. Group guitar classes can be a feasible intervention in PD and may improve mood, anxiety, and quality of life.

## 1. Introduction

Pharmacological treatments of Parkinson's Disease (PD) have been successful in reducing the motor symptoms of PD in the majority of patients, particularly during the early phases of the disease. Nevertheless, most patients who take medications still experience motor and nonmotor symptoms, and even optimal medication management does not slow the disease's progression [1].

Recent evidence suggests that music and rhythm-based interventions may provide auxiliary approaches to these pharmacologic treatments in PD patients. Music therapy has been found to improve symptoms in individuals with PD [1–3]. For example, PD patients trained to walk to music with a regular beat have reported benefits in perceptual and motor timing tasks, as well as in their ability to walk fluidly [4, 5]. An intervention relying on rhythmic music exercises (such as clapping) has also demonstrated improvements in cognition, mobility, and quality of life in PD patients [6].

Several studies suggest that training on musical instruments may also have positive effects on motor, emotional, and cognitive deficits in patients with PD. For example, neurologic music therapy (NMT) techniques, such as playing percussion instruments (e.g., conga, drums, maracas, and tambourine) (i.e., Therapeutic Instrumental Music Performance, TIMP) and rhythmic music (i.e., Rhythmic Auditory Stimulation, RAS), have been shown to enhance gait parameters and proprioception in patients with PD [7]. A small study of short-term group piano training suggested benefits for cognitive performance and psychosocial outcomes in patients with PD [8]. It has been hypothesized that music training may be beneficial because it takes advantage of the brain's functional plasticity [9]. Musical instruments can be a motivational tool to facilitate movement and induce emotional responses and social cohesion [10]. Most of the previous studies have investigated the effectiveness of percussion instruments in PD [7, 11–13]. However, to the authors' knowledge, there have been no such studies in guitar playing for the PD population. The guitar, as a portable and affordable musical instrument, provides a potential means to offer musical interventions in community settings.

The purpose of this pilot study was to investigate the feasibility and effects of using a finger style nontraditional guitar instruction program as a therapeutic approach for people with PD. We hypothesized that engagement in finger style music making on the guitar might improve functional upper extremity movements and increase participation in activities of daily living, thereby improving mood and quality of life.

## 2. Materials and Methods

### 2.1. Participants

**Figure 1:**
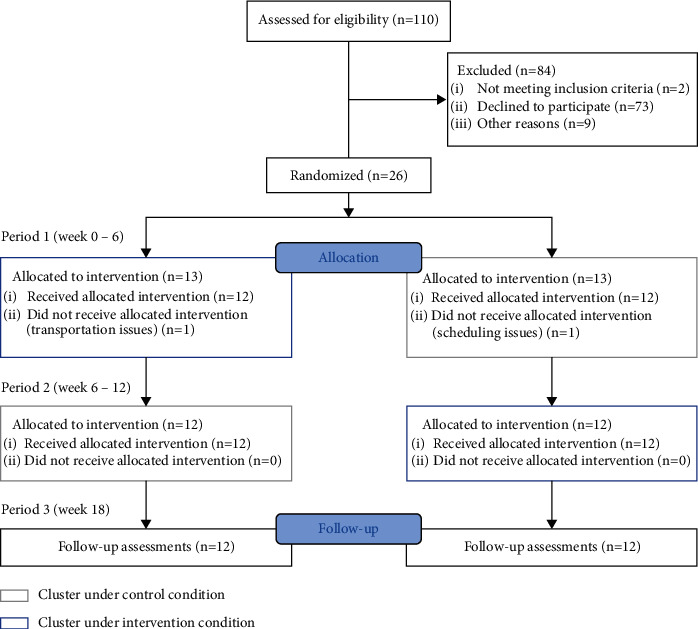
A stepped wedge cluster randomized trial consort diagram for participant screening and enrolment.

Twenty-six adult individuals with no recent experience with guitar lessons (age 67.22 ± 8.07; 17 males) with idiopathic PD diagnosis according to the UK Brain Bank Criteria [14] were enrolled in the study (Figure 1). All participants had bilateral motor symptoms at Hoehn and Yahr Stages 2–4 [15]. Inclusion criteria included the absence of another neurological disorder or injury that significantly affects the upper extremities and would preclude study participation or potentially cause participant discomfort or pain. Participants were required to score ≥17 out of 30 on the Montreal Cognitive Assessment (MoCA) [16] and to be fluent English speakers to ensure the ability to follow directions. In accordance with the declaration of Helsinki, the experimental procedures were explained to all participants and written informed consent was obtained prior to participation in the study. The study protocol was approved by the Institutional Review Board at the Johns Hopkins Medical Institutions (Baltimore, MD) and registered at ClinicalTrials.gov (NCT02925065).

### 2.2. Study Design

**Figure 2:**
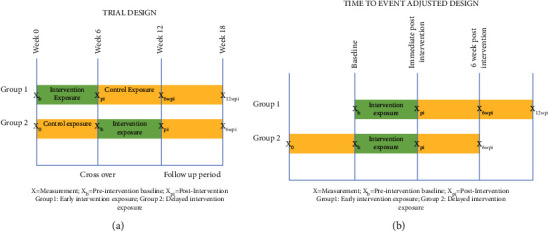
Study design. A closed cohort stepped wedge trial design with two groups (a) and a single cross-over design (b).

A closed cohort stepped wedge trial design with two groups and a single cross-over was used (Figure 2). The stepped rollout was chosen to increase logistic feasibility since (1) all interested individuals expressed a strong desire for exposure to the intervention condition and (2) simultaneous intervention implementation of all participants was prohibitive (too many participants in group guitar lessons at one time). Upon enrollment, participants were assigned to two groups using restricted randomization to balance sex and age between the groups and then the two groups were randomly assigned to early (Group 1) and late (Group 2) intervention exposure. Total trial duration was 12 weeks, with a cross-over point at 6 weeks. Group 1 experienced the intervention condition during the first 6 weeks of the trial, while Group 2 experienced it during the second 6-week period. Control condition was customary and usual treatment, while the intervention condition consisted of participation in an hour-long nontraditional fingerstyle guitar group class twice-weekly for 6 weeks in addition to customary and usual treatment. The 6-week 12-session curriculum was designed to include music that promoted finger isolation, reach and grab velocity, and eye-hand coordination timing and accuracy. Classical guitars were used, which have nylon strings and are therefore softer and easier to play than acoustic guitars. Music selections were adapted from the FJH Young Beginner Guitar Method Book 1 [17] (e.g., Olympic Bronze, Olympic Silver, Olympic Gold, Rain Rain Go Away, Hot Cross Buns, Twinkle Twinkle Little Star). Each song required a different method of fretting and plucking (See detailed curriculum descriptions in supplementary material (available (here))). The intervention was implemented by professional guitar pedagogues in a community setting at a community music school. Outcome measurements were collected during weeks 0, 6, 12, and 18. In order to limit confounding that would result from different patterns of practice activity between lessons, participants were asked not to practice between classes, and guitars were only provided during class time.

### 2.3. Study Materials

At each occasion of measurement, the following assessments were completed by assessors who were blinded to the group assignment of the participants: The Movement Disorder Society Unified Parkinson's Disease Rating Scale (MDS-UPDRS) was used to assess participants' upper extremity fine and gross motor function [15]. Interrater reliability for MDS-UPDRS motor scores was assessed by having all clinician raters (AP, SR, BMK, and ESM) independently review and rate video recordings of typical PD patients. MDS-UPDRS items included Hygiene, Handwriting, and Tremor items from Part 2 and all items from the Motor scale (Part 3). Functional manual dexterity was assessed with a typing test, where participants alternated typing letters with their middle and index fingers (“*j*” and “*k*” with the right hand, “*d*” and “*f*” with the left hand, one hand at a time) as quickly possible for 30 seconds on each hand; the accuracy was measured by the number of correctly sequenced letters participants typed [18]. Purdue Pegboard Test (PPT) [19] and Box and Block Test (BBT) [20] were also used to measure fine and gross manual dexterity, respectively (The PPT and BBT data are available from the corresponding author upon request). Self-perception of upper limb disability was assessed with the 11-item self-report Quick Disability of Arm, Shoulder, and Hand (Q-DASH) questionnaire [21]. PDQ-39 questionnaire was used to assess the quality of life on 8 dimensions of health: mobility, activities of daily living, stigma, emotional well-being, social support, cognition, communication, and physical discomfort [22]. Percentages for each dimension are calculated based on respective summed scores, where 100% is the worst health as assessed by the PDQ-39 and 0% is the best. In order to monitor the effects of mood and apathy, we used the Beck Depression Inventory-II (BDI-II) [23] as well as NeuroQoL depression and anxiety questionnaires [24, 25] and the Apathy Evaluation Scale-clinician version (AES-C) [26]. Additionally, an exit survey was administered at the end of the intervention period to assess participants' overall satisfaction with the intervention. Handedness was ascertained using the Edinburgh Handedness Inventory [27] and the Waterloo Handedness Questionnaire-Revised [28]. Feasibility was measured at the end of the trial based on an *a priori* definition of “feasibility” as completion of 9 or more sessions of the 12-session intervention program within 6 weeks.

### 2.4. Statistical Analysis

The impact of the intervention was measured using intention-to-treat analysis with a total of 26 participants (13 for each arm) to maintain the balance of risk variables between the arms at baseline [29, 30]. To determine the balance of the demographic characteristics between two groups, age, education years, and MoCA scores were compared using paired *t*-tests. In addition, Fisher's exact tests for sex, Hoehn and Yahr stages, race, and handedness were used. In order to verify consistency between the 4 MDS-UPDRS clinician raters (AP, AM, BK, SR), the intraclass correlation coefficient was calculated using their independent ratings of 6 de-identified videos of patients with idiopathic PD according to UK Brain bank criteria (none were participants in this study, and none were known to the raters; the range and degree of motor impairment of the patients in the videos were similar to those for the patients in our study) [14]. The comparisons between groups at baseline and week 6 visits used Fisher's exact tests for categorical variables and Student's *t*-tests for continuous variables.

### 2.5. Combined Group Analysis

Linear mixed effects models with a random intercept were used to estimate the changes between preintervention baseline (*X*_b_) compared with immediately after completion of intervention (*X*_pi_) and 6 weeks after intervention completion (*X*_6wpi_). All participants were combined for these analyses, regardless of whether they were randomized to an early start or delayed start, because the two groups were expected to be comparable at the same time points from completion of the guitar lessons. The indicator variable for the timeframe relative to the start of the guitar lessons was the independent variable in the regression models. Due to the small sample size, age was the only additional covariate considered.

### 2.6. Stratified Analysis by Group

Linear mixed effects models with a random intercept were used to estimate the changes from baseline at each follow-up visit for each group. The categorical indicator variable for visits was the independent variable in the models.

### 2.7. Adjustment for Multiple Comparisons

All *p* values were adjusted for multiple comparisons using the Simes method with the Stata *q*-value package [31].

## 3. Results

### 3.1. Participant Characteristics at Trial Start

A total of 26 participants were enrolled in the study. Of these, 24 (92%) completed the study. One participant from each group dropped out during the study due to transportation and scheduling challenges resulting in 12 subjects per group.

The participants ranged in age from 49 to 87, and there was no significant difference in ages between Group 1 (*M* = 67.78, SD = 7.57) and Group 2 (*M* = 66.67, SD = 8.81), *p*=0.72. There were 9 male participants in Group 1 (69.23%) and 8 participants in Group 2 (61.54%). There was also no significant difference in the years of education between Group 1 (*M* = 15.12, SD = 2.35) and Group 2 (*M* = 15.81, SD = 1.18), *p*=0.36. Further, cognitive performance using MoCA scores at the baseline was not significantly different between Group 1 (*M* = 27.46, SD = 2.18) and Group 2 (*M* = 27.08, SD = 2.90), *p*=0.66 (Table 1).

Baseline testing results revealed that 10/13 (76.92%) and 13/13 (100%) participants were categorized as Hoehn and Yahr Stage 2 in Group 1 and Group 2, respectively, while two others (15.38%) were stage 4 in Group 1. The 2 individuals classified as Hoehn and Yahr stage 4 were also the only 2 individuals who required a walker for balance assistance; as both of these individuals had normal cognitive functioning and full use of their upper extremities, they were deemed eligible for study participation. All participants were Caucasian (non-Hispanic), with the exception of one in Group 2, who was African American. Twenty-three of 26 participants (88.46%) were right-handed (Table 1).

MDS-UPDRS (Part III) Total Motor Scores ranged from 18 to 65 out of 132, with a mean of 37.8. Dyskinesias were present, per clinician rating, in 11/22 (50%) of participants (dyskinesia data were unavailable for 4 participants); however, none were reported to interfere with clinician ratings for any participant.

The intraclass correlation coefficient for interrater reliability of MDS-UPDRS motor scores based on video ratings was 0.82 (95% CI [0.53, 0.97], *p* < 0.001), indicating good interrater reliability. There were no significant differences between the two groups in outcome measures at the beginning of the trial or at the preintervention baseline. However, Group 2 MDS-UPDRS Motor scores improved from Week 0 to Week 6 (preintervention baseline), leading to a trend-level difference between the groups at preintervention baseline (corrected *p* = 0.05) (Table 2).

### 3.2. Combined Group Analysis

**Figure 3:**
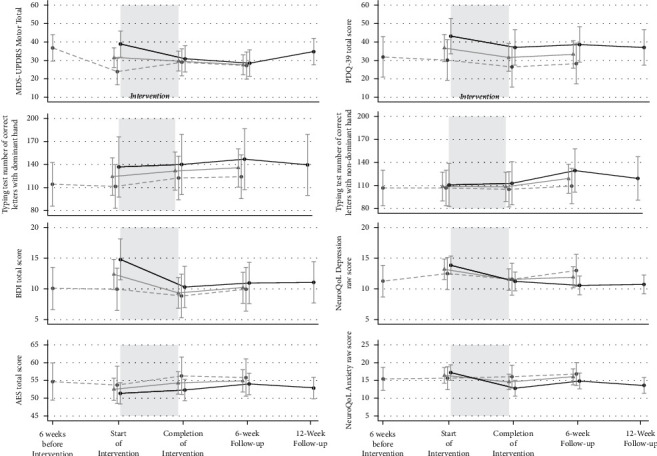
Combined and stratified analysis by intervention group-effect of intervention immediately after intervention (*X*_pi_), 6 weeks postintervention (X6wpi) and 12 weeks postintervention (X12wpi; Group 1 only).

We combined Groups 1 and 2 to compare preintervention baseline measures (*X*_b_) to those immediately following 6 weeks of guitar lessons (*X*_pi_) and measures 6 weeks after completion of guitar lessons. BDI-II scores significantly improved immediately after the completion of intervention from the preintervention assessment (corrected *p*=0.04) (Table 3). Six weeks after the completion of the intervention, MDS-UPDRS motor scores decreased by a mean of 3.8 points (95% CI [−7.4, −0.2], corrected, *p*=0.12). AES apathy score significantly improved by 2.36 points (95% CI [0.7, 4.0], corrected *p*=0.03) and PDQ-39 total quality of life scores improved by 5.19 points (95% CI [−9.4, −1.0]) immediately postintervention, though this change had only trend significance after correction for multiple comparisons (corrected *p*=0.07) (Table 3, Figure 3).

### 3.3. Stratified Analysis by Group

For the early intervention exposure group (Group 1), MDS-UPDRS total scores improved by a mean of 8.04 (95% CI [−12.4, −3.7]) points (corrected *p* = 0.004) and remained improved at 12 weeks 10.37 (95% CI [−14.7, −6.0]) points (corrected *p* < 0.001). The improvement tailed off at the final 18-week visit (corrected *p* = 0.16). This group had improvements in self-reported mood (BDI-II, corrected *p* = 0.001; NeuroQoL Depression raw score, corrected *p* = 0.02) and anxiety (Neuro-QOL Anxiety raw score, corrected *p* = 0.004) at week 6 that were still observed at week 12 and week 18 (BDI-II, both corrected *p* = 0.004; NeuroQoL Depression raw score, both corrected *p* = 0.004.; NeuroQoL Anxiety raw score, corrected *p* = 0.02 at week 18, all corrected for multiple comparisons) (Table 4, Figure 3).

The early intervention exposure group also trended towards improvement on the typing test with their nondominant hand between baseline and 6 weeks after intervention completion (95% CI [3.8, 33.9]) points (corrected *p* = 0.06), though no other changes in typing test scores were observed. There was a clinical but not a statistically significant improvement in the PDQ-39 summary index immediately postintervention (−6.21, 95% CI [−12.0, −0.5], corrected *p* = 0.12). Apathy Evaluation Scale scores for the early start group were non-significant after correction (Table 4).

Additionally, Group 2 had worsening apathy between baseline and guitar lesson starting at the borderline significance level (corrected *p* = 0.05) (Table 4). Please see the Discussion section below for potential explanations. 

Also, Group 2 showed significant improvement between week 0 and the initiation of guitar lessons (week 6), with a mean MDS-UPDRS of 36.8 (SD = 14.3) at baseline versus 23.8 (SD = 13.8) at the week 6 visit (corrected *p* = 0.003) (Table 5 in supplementary material).

### 3.4. Exit Questionnaires at the Conclusion of the Intervention

Participant responses to questionnaires administered at the end of the intervention showed that most perceptions of the intervention were positive. Ninety-five percent of participants responded “strongly agree” or “agree” to the statement that they enjoyed playing the guitar, 95% participants said they would recommend the class to friends, 95% agreed that they enjoyed the social atmosphere of the class, and 87.5% said that they would attend a class like this in the future. One participant who responded he did not enjoy playing the guitar reported that he felt unable to keep up with less impaired participants. This participant experienced a cognitive decline during this study, scoring 23/30 on the MoCA at baseline and then scoring 18/30 at the 18-week visit (the cut off for participation in this study was a score <17). In contrast, several participants indicated that they would prefer a faster-paced class. While all participants reported agreement that twice-weekly sessions were an appropriate frequency, several stated that six weeks of the class had not been long enough for them to learn much; one participant commented, “As soon as we got into it and were making progress, the lessons were over,” while another was disappointed that they “only got to learn one chord.” Compiled results of participant exit questionnaires (Table 6) are in the supplementary material (available (here)).

## 4. Discussion

The results of the intervention demonstrated significant positive effects on mood and anxiety and clinically meaningful improvements in quality of life (though the latter did not reach statistical significance after correction for multiple comparisons). The quality of life improvement was reduced 6 weeks after completion of guitar classes, suggesting that ongoing exposure to intervention is necessary to sustain improvements.

When comparing the early intervention exposure group to delayed intervention group, we found that both groups experienced improvements on the MDS-UPDRS motor scale from week 0 to week 6. There are several potential explanations for the unexpected improvements observed in the delayed start group prior to the start of guitar lessons. These include (1) placebo effect—known to be significant across PD treatment studies, which includes the possibility of delayed start participants' anticipation of improvement with upcoming lessons [32]; (2) social cues taken from the early start group during the study assessments, which were performed in the same location for both groups; (3) behavioral therapies or major life events, which were not tracked in this protocol; (4) a combination of these explanations. It seems unlikely that these differences are due to PD medication effects as all participants were assessed while taking their usual and stable medication regimens. Importantly, Group 2 participants did not report improvements in quality of life, mood, or anxiety from baseline to week 6 (prior to the start of guitar lessons), in keeping with our hypotheses.

These differences in response between groups at the week 6 visit limited our ability to draw firm conclusions from the combined group analyses. It should also be noted that the adjustments for multiple comparisons likely reduced the power to detect intervention effects in this pilot study. Further, a different proportion of Hoehn and Yahr stage in each group could have contributed to different responses (e.g., Group 1 had 3/13 (∼23%) with Hoehn and Yahr stage >2, whereas all participants in group 2 were stage 2). However, our results provide valuable information allowing for sample size calculations for future controlled clinical trials of guitar-based interventions in PD.

It is unclear whether the persistence of mood improvements in the early start group from baseline to the final week 18 assessment is attributable to the guitar lessons or to other factors, such as the ability to communicate with other study participants and the study staff regularly. The improvement in mood and quality of life (clinically and statistically significant after adjusting for multiple comparisons) scores could be attributed to several factors. One explanation would be to engage in a new activity that participants believed would be enjoyable, based on their agreement to participate. Another explanation would be the social support they received while attending the class. Several participants indicated verbally to investigators that the experience of learning in a group where others have PD was particularly meaningful.

It is notable that self-report scores, based on the Q-DASH, did not change despite the overall improvement of hand dexterity found on the MDS-UPDRS assessments, meaning that participants did not perceive a significant difference in their level of disability during routine activities. This may be due to inadequate length of intervention, relatively low levels of baseline motor impairment, or due to the fact that improvement of hand dexterity from guitar lessons has limited translatability to everyday hand function. Improvements in the BDI-II and PDQ-39 are thus unlikely to be explained by participant perception of increased functionality.

This study provides preliminary evidence that guitar lessons could be used as an active music-based intervention in PD and suggests that this intervention may potentially address PD-related motor and nonmotor symptoms simultaneously. It is the first such study known to the authors to demonstrate the feasibility of this approach. The rhythmic finger movements on the guitar represent the key difference between hand dexterity exercises and guitar lessons. Moreover, during and after the lessons, several participants expressed the desire to practice guitar outside of the class, although this was discouraged by the research team at the time due to concerns about confounding the results.

### 4.1. Limitations and Future Directions

Key limitations of this study include the small sample size and heterogeneity in the participants' baseline motor and cognitive levels; on the other hand, this heterogeneity is representative of community-dwelling individuals with PD. A larger randomized study with an active control group (e.g., a physical therapy class or a PD-specific support group meeting administered at the same frequency as the guitar-based intervention) could determine whether the improvements observed in our preliminary study can be attributed to the guitar-based intervention, as opposed to social interaction or more general physical activity. Additionally, extending postintervention follow-up would allow for a better estimate of benefit duration. To account for this, follow-up studies could use at-home tracking of guitar practice patterns through wearable sensors. Additionally, it remains to be seen whether guitar lessons can result in motor improvements that are translatable to everyday activities, such as typing.

Another goal for future research could be to examine the effects of a program that is longer in duration. Prior studies have demonstrated changes consistent with brain plasticity after at least 16–24 months of music classes, and this duration of intervention may be necessary to observe meaningful functional gains [33]. There is also a need to determine whether improvements in mood and motor function endure over longer periods of participation and whether there is a plateau effect for the program's benefits. Another modification in future studies could be to offer the program at different frequencies and intensities and allow participants a trial period to determine the pacing level most comfortable for them. This customized approach may help participants to maximally benefit, as they would be learning at a personally selected pace. Future research should also aim to address some of the difficulties in participant recruitment by offering transportation or conducting lessons at flexible hours. Also, conducting study outcome assessments for the two participant groups separately (on different days or in different spaces) would have eliminated the potential confounding from the between-group interaction. Finally, in assessing enjoyment of the lessons, it is possible that there was a self-selection effect when it came to participation and that our study population consisted of individuals particularly motivated and enthusiastic about learning the guitar. This is a common limitation to all behavioral intervention studies.

In addition, it is important to consider whether the positive effect of this study is specific to the guitar. For this, a future study could utilize another music-enriched environment in an interactive music-making group setting with an instrument similar to the guitar (e.g., ukulele).

In conclusion, while this study had a small sample size, we detected both clinically and statistically significant improvements in mood and anxiety and clinically significant improvements in overall quality of life; the quality of life improvements was reduced by 6 weeks postintervention, but the mood improvements remained significant 12 weeks after the end of the intervention. This suggests that 6 weeks of twice-per-week group guitar classes represents a feasible intervention in PD, may improve motor function, mood, and anxiety, and that some improvements may persist 6–12 weeks after concluding the guitar lessons. In the context of our delayed-start study design, between-group differences in response to guitar classes limited our ability to draw firm conclusions, but our results did provide information for sample size calculation for an appropriately powered clinical trial of a guitar-based intervention in PD with a focus on motor outcomes for future studies.

Based on our results and on feedback obtained from study participants, guitar classes may be an effective way to improve both motor and nonmotor outcomes in PD, and larger studies are warranted. The guitar is the principal instrument of choice for music therapists [34]. It is affordable and portable, with easy access to commercial instruction. If found to be beneficial, a guitar-based intervention could be readily and broadly implementable in many community-based settings worldwide.

## 5. Conclusions

This randomized pilot study investigated the feasibility and effect of guitar-based intervention in patients with PD. Our study suggests that 6 weeks of twice-per-week group guitar classes represent a feasible intervention, may improve mood, anxiety, and quality of life in PD, and that these improvements may persist 6–12 weeks after concluding the guitar lessons. Study participants found the intervention enjoyable based on their exit questionnaires. In the context of our delayed-start study design, between-group differences in response to guitar classes limited our power and ability to draw firm conclusions. However, based on our results and on feedback obtained from study participants, guitar classes may be a novel, effective way to improve both motor and nonmotor outcomes in PD, and larger studies are warranted. The clinical use of guitar playing could be an adjunct nonpharmacological therapeutic intervention in patients with PD.

## Figures and Tables

**Table 1:** Participant characteristics.

		Group 1 (n = 13)		Group 2 (n = 13)		*p*-value
		*M*	SD	*M*	SD	

Age (years)		67.78	7.57	66.67	8.81	0.72
Education (years)		15.12	2.35	15.81	1.18	0.36
MoCA (scores)		27.46	2.18	27.08	2.90	0.66
		*N*	Percentage (%)	*n*	Percentage (%)	

Sex	Male	9	69.23	8	61.54	1.00
	Female	4	30.77	5	38.46	

Hoehn and yahr	2	10	76.92	13	100	0.22
	2.5	1	7.69	0	0	
	3	0	0	0	0	
	4	2	15.38	0	0	

Race	White	13	100	12	92.31	1.00
	African-American	0	0	1	7.69	

Handedness	Right	13	100	10	76.92	0.22
	Left	0	0	3	23.08	

**Table 2:** Outcome measurements at the beginning of the trial (week 0) and at pre-intervention baseline (*X*_b_).

Motor and emotion outcomes, mean (SD)	Trial start (week 0)				Preintervention baseline (*X*_*b*_)			
	Group1 (*n* = 13)	Group 2 (*n* = 13)	Uncorrected *p*-value	Corrected *p*-value^a^	Group1 (*n* = 13)	Group 2 Week 6 visit (*n* = 13)	Uncorrected *p*-value	Corrected *p*-value^a^
MDS-UPDRS motor total	38.8 (13.6)	36.8 (14.3)	0.71	0.80	38.8 (13.6)	23.8 (13.8)	0.01^∗∗^	0.05
Typing test accuracy, dominant hand	137.0 (78.3)	114.4 (53.2)	0.40	0.53	137.0 (78.3)	111.7 (46.4)	0.33	0.47
Typing test accuracy, non-dominant hand	110.6 (49.2)	106.5 (42.6)	0.82	0.85	110.6 (49.2)	106.6 (42.2)	0.83	0.85
PDQ39 total score	43.2 (20.4)	31.8 (23.0)	0.19	0.32	43.2 (20.4)	30.2 (19.7)	0.11	0.22
Beck depression inventory total score	14.8 (6.3)	10.1 (6.2)	0.07	0.16	14.8 (6.3)	9.9 (7.3)	0.08	0.17
Apathy evaluation scale total score	51.3 (6.6)	55.2 (10.0)	0.26	0.40	51.3 (6.6)	53.7 (11.8)	0.05	0.13
NeuroQoL depression raw score	13.8 (4.2)	11.2 (2.9)	0.08	0.17	13.8 (4.2)	12.5 (6.0)	0.50	0.64
NeuroQoL anxiety raw score	17.2 (5.0)	15.4 (4.4)	0.35	0.50	17.2 (5.0)	15.6 (6.7)	0.52	0.64

^a^ *p* values are adjusted for multiple comparisons using the Simes method. ^∗^*p* < 0.05. ^∗∗^*p* < 0.01.

**Table 3:** Combined group analysis-effect of intervention immediately after and 6 weeks after completion of the intervention (*n* = 26).

Outcomes	Changes from preintervention (X_b_) to immediately postintervention (X_pi_)				Changes from preintervention (X_b_) to 6 weeks after completion of intervention			
	Coefficient	95% CI	Uncorrected *p*-value	Corrected *p*-value^a^	Coefficient	95% CI	Uncorrected *p*-value	Corrected *p*-value^a^
MDS-UPDRS motor total	−1.76	−5.4, 1.9	0.34	0.50	−3.80	−7.4, −0.2	0.04^∗^	0.12
Typing test accuracy with dominant hand	7.19	−5.6, 20.0	0.27	0.41	11.36	−1.4, 24.1	0.08	0.17
Typing test accuracy with nondominant hand	−0.43	−10.6, 9.8	0.93	0.95	10.16	0.0, 20.3	0.05	0.13
PDQ-39 total score	−5.19	−9.4, −1.0	0.02^∗^	0.07	−3.48	−7.7, 0.7	0.10	0.21
Beck depression Inventory-II total score	−3.04	−5.2, −0.9	0.006^∗∗^	0.04*∗*	−2.16	−4.3, 0.0	0.05	0.13
Apathy evaluation scale total score	1.74	0.1, 3.4	0.04^∗^	0.12	2.36	0.7, 4.0	0.005^∗∗^	0.03^∗^
NeuroQoL depression raw score	−1.65	−2.9, −0.4	0.01^∗^	0.05	−1.27	−2.5, 0.0	0.049^∗^	0.13
NeuroQoL anxiety raw score	−1.83	−3.6, −0.1	0.04^∗^	0.12	−0.41	−2.2, 1.4	0.65	0.77

^a^ *p* values are adjusted for multiple comparisons using the Simes method. ^∗^*p* < 0.05. ^∗∗^*p*  < 0.01.

**Table 4:** Stratified analysis by intervention groups-effect of intervention immediately after intervention (*X*_pi_), 6 weeks postintervention (*X*_6wpi_), and 12 weeks postintervention (*X*_12wpi_; Group 1 only).

Outcomes	Time point	Early intervention exposure group-Group 1 (*n* = 13)					Delayed intervention exposure group-Group 2 (*n* = 13)				
		Mean (SD)	Coefficient	95% CI	Uncorrected *p*-value	Corrected *p*-value	Mean (SD)	Coefficient	95% CI	Uncorrected *p*-value	Corrected *p*-value
MDS-UPDRS motor total	Postintervention (X_pi_)	29.8 (11.3)	−8.04	−12.4, −3.7	<0.001^∗∗∗^	0.004^∗∗^	27.4 (15.0)	5.06	0.74, 9.38	0.02^*∗*^	0.09
	X_6wpi_	27.5 (16.2)	−10.37	−14.7, −6.0	<0.001^∗∗∗^	<0.001^∗∗^	25.7 (11.0)	3.31	−1.01, 7.63	0.13	0.24
	X_12wpi_	33.8 (12.5)	−4.04	−8.4, 0.3	0.07	0.16					

Typing accuracy, dominant hand	Postintervention (X_pi_)	144.8 (72.1)	3.05	−14.2, 20.3	0.73	0.80	123.8 (54.0)	10.97	−4.30, 26.25	0.16	0.29
	X_6wpi_	151.8 (74.4)	10.05	−7.2, 27.3	0.25	0.40	125.1 (64.9)	12.31	−2.97, 27.58	0.11	0.22
	X_12wpi_	144.4 (78.3)	2.63	−14.6, 19.8	0.76	0.82					

Typing accuracy, nondominant hand	Postintervention (X_pi_)	115.0 (53.8)	2.11	−12.94, 17.15	0.78	0.83	106.6 (42.2)	−1.93	−12.78, 8.92	0.73	0.80
	X_6wpi_	131.8 (63.7)	18.86	3.8, 33.9	0.01	0.06	111.0 (51.5)	2.49	−8.36, 13.34	0.65	0.77
	X_12wpi_	121.6 (49.4)	8.69	−6.35, 23.83	0.26	0.40					

PDQ-39 total score	Postintervention (X_pi_)	34.7 (14.7)	−6.21	−12.0, −0.5	0.03	0.12	24.2 (18.4)	−3.72	−9.35, 1.90	0.19	0.32
	X_6wpi_	36.3 (14.7)	−4.63	−10.4, 1.1	0.12	0.22	26.0 (21.4)	−1.89	−7.51, 3.73	0.51	0.64
	X_12wpi_	34.7 (18.1)	−6.17	−11.9, −0.4	0.04*∗*	0.12					

Beck depression inventory–II total score	Postintervention (X_pi_)	9.2 (6.6)	−4.50	−6.6, −2.4	<0.001^∗∗∗^	0.001^∗∗^	8.8 (5.3)	−1.08	−4.13, 1.97	0.49	0.63
	X_6wpi_	9.8 (3.0)	−3.83	−5.9, −1.7	<0.001^∗∗∗^	0.004^∗∗^	9.8 (7.6)	0.01	−3.04, 3.06	0.10	0.10
	X_12wpi_	9.9 (5.1)	−3.75	−5.8, −1.7	<0.001^∗∗∗^	0.004^∗∗^					

Apathy evaluation scale total score	Postintervention (X_pi_)	52.7 (6.1)	0.98	−1.3, 3.3	0.40	0.53	56.8 (8.4)	2.53	0.63, 4.43	0.009^∗∗^	0.05
	X_6wpi_	54.3 (4.6)	2.65	0.3, 5.0	0.03^*∗*^	0.10	56.3 (9.9)	2.12	0.22, 4.02	0.029^*∗*^	0.11
	X_12wpi_	53.3 (5.0)	1.57	−0.7, 3.9	0.18	0.32					

NeuroQoL depression raw score	Postintervention (X_pi_)	11.1 (2.5)	−2.64	−4.4, −0.9	0.003^*∗*^	0.02^*∗*^	11.3 (4.2)	−0.90	−2.71, 0.91	0.33	0.47
	X_6wpi_	10.4 (1.9)	−3.31	−5.0, −1.6	<0.001^∗∗∗^	0.004^∗∗^	12.8 (6.1)	0.52	−1.29, 2.33	0.58	0.70
	X_12wpi_	10.6 (1.9)	−3.14	−4.9, −1.4	<0.001^∗∗∗^	0.004^∗∗^					

NeuroQoL anxiety raw score	Postintervention (X_pi_)	12.3 (2.5)	−4.42	−6.8, −2.1	<0.001^∗∗∗^	0.004^∗∗^	16.1 (6.0)	0.40	−1.89, 2.68	0.74	0.80
	X_6wpi_	14.4 (3.7)	−2.33	−4.7, 0.0	0.05	0.13	16.8 (7.6)	1.15	−1.14, 3.43	0.33	0.50
	X_12wpi_	13.2 (4.1)	−3.58	−5.9, −1.2	0.003^∗∗^	0.02^*∗*^					

^1^ *p* values are adjusted for multiple comparisons using the Simes method. *∗p* < 0.05. ∗∗*p*  < 0.01. ∗∗∗*p* < 0.001.

## Data Availability

The data used to support the findings of this study are available from the corresponding author upon reasonable request.

## Supplementary Materials

Supplementary Materials 1. Supplementary_File1: Curriculum description and Score example. 2. Supplementray_File2: A. Table 5: Comparison of motor and emotion outcomes between the time of randomization (Baseline) and the initiation of the guitar lessons (Week 6) for the delayed start group. B. Table 6: Compiled results of participant exit questionnaires. 3. Supplementary_File3: A. Baseline Characteristics of MDS-UPDRS, Q-DASH, NeuroQoL, and PDQ-39 (Table a–d).
