# Supplementary material for: GuitarPD: A Randomized Pilot Study on the Impact of Nontraditional Guitar Instruction on Functional Movement and Well-Being in Parkinson's Disease
Source: Parkinsons Dis. 2022 Jun 25;2022:1061045. doi: 10.1155/2022/1061045 (PMC9252755; doi:10.1155/2022/1061045)
Supplement: Supplementary Materials — 1. Supplementary_File1: Curriculum description and Score example. 2. Supplementray_File2: A. Table 5: Comparison of motor and emotion outcomes between the time of randomization (Baseline) and the initiation of the guitar lessons (Week 6) for the delayed start group. B. Table 6: Compiled results of participant exit questionnaires. 3. Supplementary_File3: A. Baseline Characteristics of MDS-UPDRS, Q-DASH, NeuroQoL, and PDQ-39 (Table a–d). [file 1061045.f1.zip › 1061045.f1/Guitar_PD_Supplementary_File1_Curriculum description and score example.pdf]

Curriculum Description

The curriculum was developed by breaking down the fundamental components of playing the guitar: technical movements of both the left and right hands as well as basic musical concepts for the participants.

The program was structured to develop the following:

- Seating and positioning with the guitar
- Basic right hand setup/positioning
- Established contact points for right hand fingers
- Right hand alternation (between index & middle fingers)
- Right hand string crossing
- Basic left hand setup
- Development of left hand finger placement (contact points of fingertips)
- Development of left hand finger independence: fretting the string from an open string position , left hand finger combinations: 1-3, 1-2, 1-4, 2-4, 2-1
- Development of left hand horizontal shifts.

This graded curriculum first developed the right-hand technique of participants (the hand that generates sound from the guitar) using the open strings of the instrument and elementary rhythms to establish a consistent pulse. With the basic right-hand mechanics in place, elements of the left hand (which controls the overall pitch of the instrument) were then introduced. The introduction of the left-hand fingers was done individually with a short "piece" created for each individual finger. Additionally, there were pieces that created combined left-hand patterns to establish new physical challenges within a musical context.

Note: The repertoire chosen to develop these physical skills used small musical studies that worked on a particular physical skill within a musical context/accompaniment provided by the instructor.

Score examples

The material below was adapted and modified for the study from Adapted from "*The FJH Young Beginner Guitar Method Lesson Book 1*" FJH music company, Inc.

Olympic Bronze

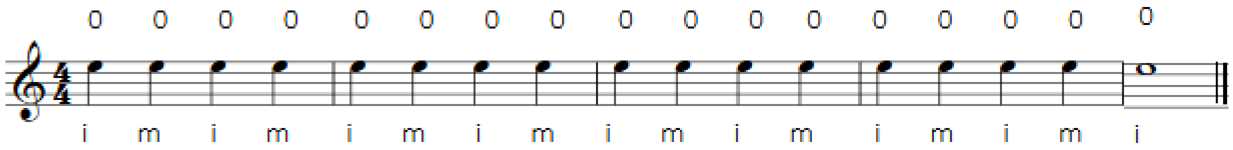

Olympic Silver

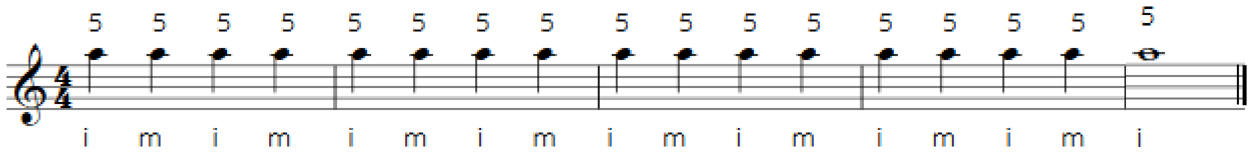

Olympic Gold

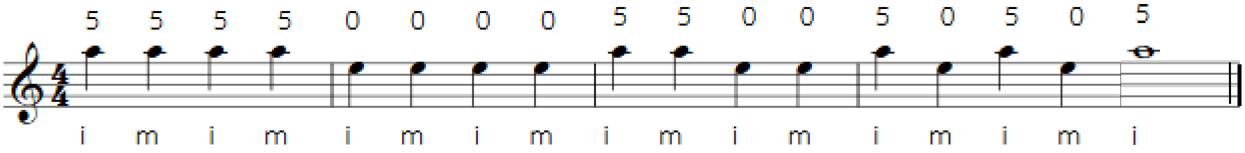

Rain, Rain, Go Away

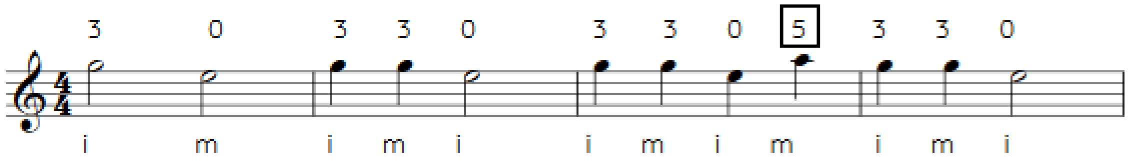

Hot Cross Buns

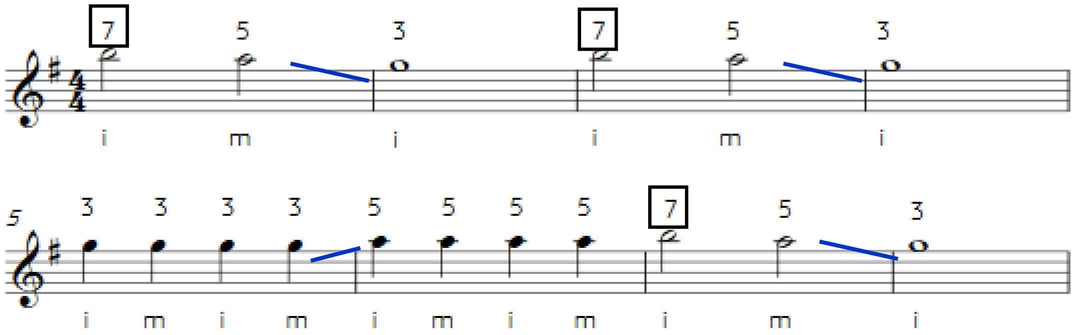

Twinkle, Twinkle, Little Star

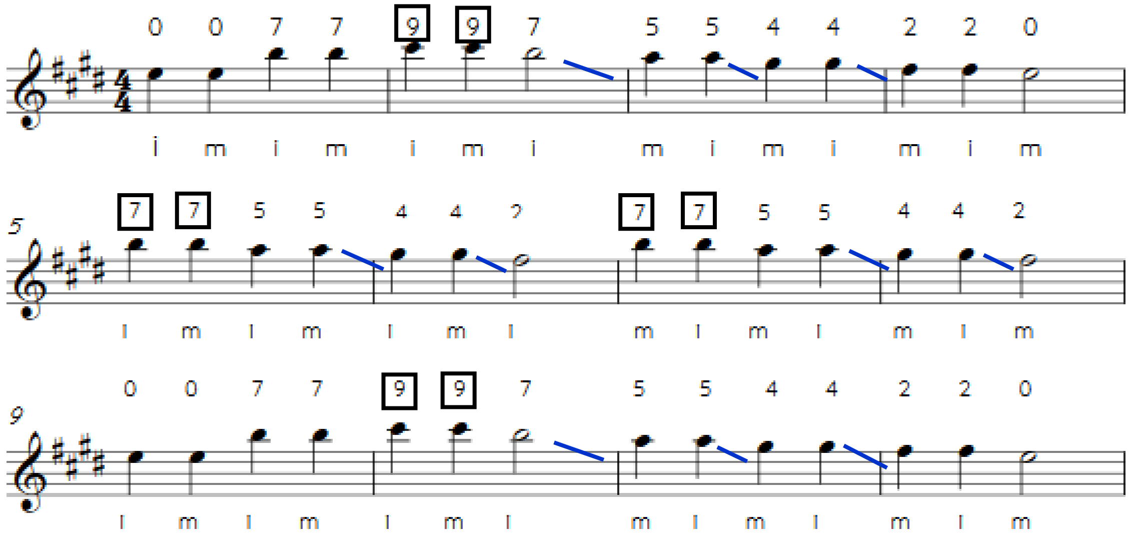

- All notes are to be played on the high E string only (1st String)
- Numbers indicate which fret to be played in the music (0 means open string, no fretting)
- All notes are played with index finger of the fretting (left) hand, unless fret number is surrounded by a square. Square indicates ring finger.
- Letter ‘i’ indicates the index finger of the plucking (right hand) and ‘m’ is for middle finger.
- ‘ ’ or ‘ ’ indicates the index finger sliding between frets.

- <u>Olympic Bronze:</u> alternating index and middle fingers of the plucking hand (right hand) on the first open string (no fretting hand involvement).
- <u>Olympic Silver:</u> Index finger of fretting hand is held down on 5th fret.
- <u>Olympic Gold:</u> Index finger of fretting hand practices placing onto and lifting off of the string.
- <u>Rain Rain Go Away:</u> Ring finger of fretting hand is introduced and used in combination with index finger. Different rhythms are introduced (half notes and quarter notes)
- <u>Hot Cross Buns:</u> Both index and ring fingers of the fretting hand. Index finger practices shifting between frets 3 and 5 by sliding over rather than lifting. Different rhythms are introduced (whole notes).
- <u>Twinkle Twinkle Little Star:</u> All accumulated skills from previous songs are put to use (plucking hand alternating, fretting hand using two fingers, shifting, and lifting). Index finger practices shifting between frets 2 and 4, 4 and 5, as well as 5 and 7 by sliding over rather than lifting.
- <u>Strumming Practice</u>: This involved the fretting hand holding down multiple fingers at the same time on different strings/frets while the plucking hand practiced strumming
