# Supplementary material for: GuitarPD: A Randomized Pilot Study on the Impact of Nontraditional Guitar Instruction on Functional Movement and Well-Being in Parkinson's Disease
Source: Parkinsons Dis. 2022 Jun 25;2022:1061045. doi: 10.1155/2022/1061045 (PMC9252755; doi:10.1155/2022/1061045)
Supplement: Supplementary Materials — 1. Supplementary_File1: Curriculum description and Score example. 2. Supplementray_File2: A. Table 5: Comparison of motor and emotion outcomes between the time of randomization (Baseline) and the initiation of the guitar lessons (Week 6) for the delayed start group. B. Table 6: Compiled results of participant exit questionnaires. 3. Supplementary_File3: A. Baseline Characteristics of MDS-UPDRS, Q-DASH, NeuroQoL, and PDQ-39 (Table a–d). [file 1061045.f1.zip › 1061045.f1/Guitar_PD_Supplementary_File2_Table 5 and Table 6.pdf]

Table 5: Comparison of motor and emotion outcomes between the time of randomization (Baseline) and the initiation of the guitar lessons (Week 6) for the delayed start group

Motor and emotion outcomes, mean (SD)	Delayed start group			
	Baseline visit (n=13)	Week 6 visit (n=13)	Uncorrected *P*-value	Corrected *P*-value[1]
MDS-UPDRS Motor Total	36.8 (14.3)	23.8 (13.8)	<.001***	.003**
Typing test accuracy with dominant hand	114.4 (53.2)	111.7 (46.4)	.579	.731
Typing test accuracy with non-dominant hand	106.5 (42.6)	106.6 (42.2)	.982	.996
PDQ39 total score	31.8 (23.0)	30.2 (19.7)	.627	.778
Beck Depression Inventory total score	10.1 (6.2)	9.9 (7.3)	.914	.953
Apathy Evaluation Scale total score	55.2 (10.0)	53.7 (11.8)	.413	.561
NeuroQoL Depression raw score	11.2 (2.9)	12.5 (6.0)	.308	.481
NeuroQoL Anxiety raw score	15.4 (4.4)	15.6 (6.7)	.813	.868

[1]*P* values are adjusted for multiple comparisons using the Simes meth

**$p < .01$

*** $p < .001$

Table 6: Compiled results of participant exit questionnaires

	Strongly Agree	Agree	Disagree	Strongly Disagree
I enjoyed playing the guitar	75%<br>18/24	20.83%<br>5/24	0%<br>0/24	4.17%<br>1/24[a]
I was able to follow instructions	62.5%<br>15/24	25%<br>6/24	12.5%<br>3/24[b]	0%<br>0/24
I enjoyed the social atmosphere of the class	66.67%<br>16/24	29.17%<br>7/24	4.17%<br>1/24[c]	0%<br>0/24
It was convenient to attend these classes	45.83%<br>11/24	33.33%<br>8/24	16.67%<br>4/24[d]	4.17%<br>1/24[d]
This class helped my arms & hands move better	27.27%<br>6/22*	50%<br>11/22*	22.73%<br>5/22*	0%<br>0/22*
I would recommend this class to my friends	79.17%<br>19/24	16.67%<br>4/24	4.17%<br>1/24[e]	0%<br>0/24
I would attend a class like this in the future	75%<br>18/24	12.5%<br>3/24	8.33%<br>2/24[f]	4.17%<br>1/24[f]

	Yes	No	Maybe
Was the frequency of classes (2x/wk) appropriate?	100%<br>24/24	0%<br>0/24	0%<br>0/24
Are you planning to seek music instruction as a recreational activity?	79.17%<br>19/24	16.67%<br>4/24	4.17%<br>1/24
Would you like to be notified of similar research projects that involve music instruction?	87.5%<br>21/24	8.33%<br>2/24	0%<br>0/24

*one participant responded “I don’t know” and another left it blank
