# Supplementary material for: GuitarPD: A Randomized Pilot Study on the Impact of Nontraditional Guitar Instruction on Functional Movement and Well-Being in Parkinson's Disease
Source: Parkinsons Dis. 2022 Jun 25;2022:1061045. doi: 10.1155/2022/1061045 (PMC9252755; doi:10.1155/2022/1061045)
Supplement: Supplementary Materials — 1. Supplementary_File1: Curriculum description and Score example. 2. Supplementray_File2: A. Table 5: Comparison of motor and emotion outcomes between the time of randomization (Baseline) and the initiation of the guitar lessons (Week 6) for the delayed start group. B. Table 6: Compiled results of participant exit questionnaires. 3. Supplementary_File3: A. Baseline Characteristics of MDS-UPDRS, Q-DASH, NeuroQoL, and PDQ-39 (Table a–d). [file 1061045.f1.zip › 1061045.f1/Guitar_PD_Supplementary_File3_Baseline Characteristics_revised.pdf]

Table a: Baseline MDS-UPDRS severity scores for all participants (higher scores reflect a greater level of disability)

	Symptom	Normal	Slight	Mild	Moderate	Severe
Selected MDS-UPDRS ADL Items	Hygiene	11/26 (42.31%)	8/26 (30.77%)	7/26 (26.92%)	0	0
	Handwriting	5/26 (19.23%)	6/26 (23.08%)	5/26 (19.23%)	7/26 (26.92%)	3/26 (11.54%)
	Tremor	7/26 (26.92%)	8/26 (30.77%)	5/26 (19.23%)	6/26 (23.08%)	0
MDS-UPDRS Rigidity Items	Rigidity – neck	1/26 (3.85%)	10/26 (38.46%)	10/26 (38.46%)	3/26 (11.54%)	4/26 (15.39%)
	Rigidity – Right Upper Extremity	6/26 (23.08%)	8/26 (30.77%)	12/26 (46.15%)	0	0
	Rigidity – Left Upper Extremity	7/26 (26.92%)	8/26 (30.77%)	10/26 (38.46%)	1/26 (3.85%)	0
	Rigidity – Right Lower Extremity	7/26 (26.92%)	6/26 (23.08%)	11/26 (42.31%)	2/26 (7.69%)	0
	Rigidity – Left Lower Extremity	6/26 (23.08%)	3/26 (11.54%)	13/26 (50%)	4/26 (15.39%)	0
MDS-UPDRS Agility Items	Finger tapping – Right Hand	2/26 (7.69%)	8/26 (30.77%)	9/26 (34.62%)	7/26 (26.92%)	0
	Finger tapping – Left Hand	0	4/26 (15.39%)	13/26 (50%)	8/26 (30.77%)	0
	Hand movements - Right Hand	4/26 (15.39%)	8/26 (30.77%)	11/26 (42.31%)	3/26 (11.54%)	0
	Hand movements - Left Hand	0	10/26 (38.46%)	9/26 (34.62%)	7/26 (26.92%)	0
	Pronation-Supination - Right Hand	3/26 (11.54%)	10/26 (38.46%)	8/26 (30.77%)	5/26 (19.23%)	0
	Pronation-Supination -	1/26 (3.85%)	5/26 (19.23%)	13/26 (50%)	7/26 (26.92%)	0

	Left Hand					
	Toe Tapping – Right foot	3/26 (11.54%)	9/26 (34.62%)	8/26 (30.77%)	5/26 (19.23%)	1/26 (3.85%)
	Toe Tapping – Left foot	1/26 (3.85%)	9/26 (34.62%)	8/26 (30.77%)	6/26 (23.08%)	2/26 (7.69%)
	Leg Agility – Right leg	10/26 (38.46%)	4/26 (15.39%)	10/26 (38.46%)	2/26 (7.69%)	0
	Leg Agility – Left leg	5/26 (19.23%)	10/26 (38.46%)	7/26 (26.92%)	4/26 (15.39%)	0
MDS-UPDRS Mobility/Gait Items	Arising from chair	18/26 (69.23%)	5/26 (19.23%)	2/26 (7.69%)	1/26 (3.85%)	0
	Gait	6/26 (23.08%)	13/26 (50%)	4/26 (15.39%)	3/26 (11.54%)	0
	Freezing of Gait	25/26 (96.15%)	0	1/26 (3.85%)	0	0
	Postural Stability	22/26 (84.62%)	2/26 (7.69%)	1/26 (3.85%)	1/26 (3.85%)	0
	Posture	6/26 (23.08%)	13/26 (50%)	4/26 (15.39%)	3/26 (11.54%)	0
	Global spontaneity of movement	7/26 (26.92%)	12/26 (46.15%)	4/26 (15.39%)	3/26 (11.54%)	0
MDS-UPDRS Tremor items	Postural tremor – right hand	12/26 (46.15%)	12/26 (46.15%)	2/26 (7.69%)	0	0
	Postural tremor – left hand	9/26 (34.62%)	13/26 (50%)	4/26 (15.39%)	0	0
	Kinetic tremor – right hand	10/26 (38.46%)	12/26 (46.15%)	4/26 (15.39%)	0	0
	Kinetic tremor – left hand	3/26 (11.54%)	14/26 (53.85%)	9/26 (34.62%)	0	0
	Rest tremor amplitude – right upper extremity	11/26 (42.31%)	6/26 (23.08%)	7/26 (26.92%)	1/26 (3.85%)	1/26 (3.85%)
	Rest tremor amplitude – left upper extremity	16/26 (61.54%)	5/26 (19.23%)	3/26 (11.54%)	2/26 (7.69%)	0
	Rest tremor	21/26	4/26	1/26	0	0

amplitude – right lower extremity	(80.77%)	(15.39%)	(3.85%)		
Rest tremor amplitude – left lower extremity	24/26 (92.31%)	2/26 (7.69%)	0	0	0
Rest tremor amplitude – lip/jaw	23/26 (88.46%)	2/26 (7.69%)	0	1/26 (3.85%)	0
Constancy of rest tremor	8/26 (30.77%)	8/26 (30.77%)	6/26 (23.08%)	3/26 (11.54%)	1/26 (3.85%)

Table b: Q-DASH – scores range from 0-100, with higher scores indicating greater disability

	**Score: 0-20**	**Score: 21-40**	**Score: 41-60**	**Score: 61-80**	**Score: 81-100**
Q-DASH	19/26 (73.08%)	3/26 (11.54%)	4/26 (15.39%)	0	0

Table c: NeuroQoL: raw scores range from 8-40 with lower scores indicating greater disability. T-scores have a mean of 50 and SD of 10 with lower scores indicating greater disability

**T-score**	**T-Score: 20-29**	**T-Score: 30-39**	**T-Score: 40-49**	**T-Score: 50-60**
Neuro-QoL Upper Extremity	2/26 (7.69%)	11/26 (42.31%)	7/26 (26.92%)	6/26 (23.08%)
Neuro-QoL Lower Extremity	0	9/26	11/26 (42.31%)	6/26 (23.08%)

Table d: Baseline Parkinson's Disease Questionnaire-39 Results

NB: Higher scores reflect greater disability.

PDQ-39	Minimum Score	Maximum Score	Mean Score
Mobility	0	30	10.19
Adaptive Living	0	16	5.58
Emotional Well-Being	0	14	5.27
Stigma	0	13	1.73
Social Support	0	5	1.77
Cognition	0	12	5.04
Communication	0	8	2.88
Bodily Discomfort	1	12	5.00
Total	6	83	37.46
